# A Randomized Study on Postrelapse Disease-Free Survival with Adjuvant Mistletoe versus Oral Etoposide in Osteosarcoma Patients

**DOI:** 10.1155/2014/210198

**Published:** 2014-03-31

**Authors:** Alessandra Longhi, Marcus Reif, Erminia Mariani, Stefano Ferrari

**Affiliations:** ^1^Musculoskeletal Oncology Department, Istituto Ortopedico Rizzoli, Via Pupilli 1, 40136 Bologna, Italy; ^2^Institute for Clinical Research, Hardenbergstraße 20, 10623 Berlin, Germany; ^3^Immunorheumatology and Tissue Regeneration Laboratory, Istituto Ortopedico Rizzoli, Via Barbiano 1/10, 40136 Bologna, Italy

## Abstract

*Background*. Osteosarcoma is a highly malignant bone tumour. After the second relapse, the 12-month postrelapse disease-free survival (PRDFS) rate decreases below 20%. Oral Etoposide is often used in clinical practice after surgery as an “adjuvant” outside any protocol and with only limited evidence of improved survival. *Viscum album fermentatum Pini* (*Viscum*) is an extract of mistletoe plants grown on pine trees for subcutaneous (sc) injection with immunomodulatory activity. *Methods*. Encouraged by preliminary findings, we conducted a study where osteosarcoma patients free from disease after second metastatic relapse were randomly assigned to *Viscum* sc or Oral Etoposide. Our goal was to compare 12-month PRDFS rates with an equivalent historical control group. *Results*. Twenty patients have been enrolled, with a median age of 34 years (range 11–65) and a median follow-up time of 38.5 months (3–73). The median PRDSF is currently 4 months (1–47) in the Etoposide and 39 months (2–73) in the *Viscum* group. Patients getting *Viscum* reported a higher quality of life due to lower toxicity. *Conclusion*. *Viscum* shows promise as adjuvant treatment in prolonging PRDFS after second relapse in osteosarcoma patients. A larger study is required to conclusively determine efficacy and immunomodulatory mechanisms of *Viscum* therapy in osteosarcoma patients.

## 1. Introduction

Osteosarcoma is an aggressive malignant neoplasm, for which neoadjuvant chemotherapy with the four most effective drugs (Doxorubicin, Methotrexate, Cisplatin, and Ifosfamide) has improved 5-year disease-free survival (DFS) rate from 10% to 60%. Treatment for patients who relapse, either locally or with distant metastases mainly in lungs, is primarily surgical. The prognosis is poor, with long-term postrelapse survival <20%. Yet, most of the patients relapse a second time, mainly in the lung (40%; see [1]). Fagioli et al. [2] reported a 3-year DFS rate of 12% after the second relapse in patients who received surgery and chemotherapy, with 80% of patients rerelapsing within 1 year. In the study of Bacci et al. [3] on 235 osteosarcoma patients who relapsed after neoadjuvant chemotherapy, 120 (51%) patients had a second relapse with a median interval between second and third relapse of 11.8 months. Of these 120 patients only 14 (11.6%) achieved prolonged remission. The role of second-line chemotherapy for recurrent osteosarcoma is much less well defined than that of surgery, and there is no accepted standard regimen.

Besides surgery, different drugs have been employed (Ifosfamide, Cyclophosphamide, Etoposide, Methotrexate, Vinorelbine, and Gemcitabine plus Docetaxel) as “adjuvant” treatment postsurgery and eventually as monotherapy for unresectable disease, yet with scarce results [4]. A recent retrospective analysis (July 2013) on 110 relapsed osteosarcoma patients from St. Jude Hospital confirmed that surgery at relapse is essential for survival and chemotherapy may only slow disease progression in patients without second complete remission [5].

Etoposide is a topoisomerase II inhibitor mainly used intravenously in the treatment of several tumors (i.e., lymphomas, lung cancer, ovarian cancer, and Ewing's sarcoma), both in combination and in monotherapy. Some osteosarcoma protocols employ Etoposide iv in neoadjuvant schemes used for poor responders in postoperative treatment with intensification purpose (i.e., [6]). In the only osteosarcoma study on Oral Etoposide 50 mg/m^2^/daily for 14 days as monotherapy, Kebudi et al. [7] reported a 15% response rate (RR) in relapsed pediatric patients. Sandri et al. [8] reported a successful use of Oral Etoposide at 50 mg/m^2^ in children with recurrent ependymomas, showing a 40% RR. Oral Etoposide is well tolerated, with only mild adverse drug reactions like nausea, leucopenia, and alopecia. Yet, hematologic toxicity is one of the main limiting toxicities in second- or third-line chemotherapy in these heavily pretreated patients. Etoposide cannot be given for a longer period due to the risk of secondary hematologic malignancy.

Mistletoe therapy is widely used in cancer patients (>60% of tumor patients in Germany and Switzerland). It is derived from* Viscum album* L., a semiparasitic plant, which is classified into three subspecies (*deciduous tree mistletoe*,* pine mistletoe*, and* fir mistletoe*) each having its particular host tree (apple tree, oak, elm, pine, and fir). Its effects are similar to those of other biological response modifiers in targeting the immune system [9–15], (review in [16]). Main components of the whole plant extract are mistletoe lectins I, II, and III and six viscotoxins and polysaccharides. Lectins have shown cytostatic and immunomodulatory activity* in vitro* while viscotoxins have been reported to have cytoxic activity. Polysaccharides have shown immunomodulatory activities, that is, an increase in NK activity [17, 18]. The total plant extract is an immunostimulant (increase of NK cells, T-lymphocytes, and macrophages) and has apoptotic activity* in vitro* [19] and* in vivo* [20].

*Viscum album fermentatum Pini* (*Viscum*, equivalent to Iscador P, Weleda AG, Arlesheim, Switzerland) is one of several commercial* Viscum album* preparations and on the market since 1917. Administered as subcutaneous injection it is locally and systemically well tolerated.* Viscum* preparations have experimentally also been applied intravenously [21] but the subcutaneous injection is the only licensed form of application (exempt from the homeopathically potentiated mistletoe extracts ABNOBAviscum D6 to D30).

The study reported here examines the postrelapse disease-free survival (PRDFS) in patients at high risk for further relapse after surgery for a second relapse receiving either Etoposide or* Viscum*. Our aim is to compare the 12-month PRDFS rate of each of the two study arms with a historical cohort of patients. A preliminary report of the first ten patients of this study has already been published earlier [22]. Here, we present the clinical results for all patients.

## 2. Materials and Methods

This is a prospective, randomized, open-label study conducted according to the Declaration of Helsinki and approved by the ethics committee of the Istituto Ortopedico Rizzoli (IOR), Bologna, and by the Italian Competent Authorities. All patients provided written informed consent before study entry. The study is registered in the in the EU clinical trials register, EudraCT number 2006-002676-18.

### 2.1. Patients

Inclusion criteria comprised the histologically confirmed diagnosis of osteosarcoma or spindle cell sarcoma of the bone after a second relapse; absence of metastases and local relapse after surgery; age ≥10 years; ECOG ≤2; adequate bone marrow function (i.e., peripheral absolute neutrophils >1500, platelets >100.000); and further lab parameters restricted to bilirubin <2, creatinine < 1.5x normal, and a signed informed consent. Exclusion criteria were bone sarcomas of other histological type or any other malignancy prior to study; missing staging criteria; last antineoplastic treatment received within 30 days prior to study entry; treatment with Etoposide or* Viscum album* extract prior to study entry; concomitant treatment with drugs having either immunostimulatory or immunosuppressive properties; pregnancy.

### 2.2. Patient Assignment

Patients were randomized 1 : 1 to receive either Etoposide or* Viscum*. Randomization was requested by fax and performed centrally at the Institute for Clinical Research, Berlin, according to an unrestricted randomization list created by a statistician from the IOR uninvolved in any further aspect of the trial.

### 2.3. Study Medication and Treatment

The* Viscum album* extract applied in this study is an approved drug and has a marketing authorization under the name “Iscador P” (Weleda AG, Arlesheim, Switzerland) in Germany, Switzerland, and Austria. In Italy, it is registered as homeopathic remedy under the name* Viscum album fermentatum Pini*. According to the manufacturer, this mistletoe extract contained mistletoe lectins of up to 40 ng/mL in the 20 mg dose of Series II (personal communication). Immunomodulatory activity of this preparation has previously been demonstrated regarding an increase in TNF-*α* and IL-6 [23], natural killer (NK) cell cytotoxicity [24], activation of CD4^+^ T-helper cells and monocytes [25], maturation of dendritic cells [26], and activation of macrophages [27]. In the actual study, immunological parameters including NK T lymphocytes, IL-2, IL-4, IL-12, IL-15, *γ*-IFN, and IP-10 were determined quarterly yet will be presented elsewhere.

*Viscum album* extract was injected subcutaneously (abdominal) 3 times/week. Starting dose was 2 boxes of Series 0 (0.01, 0.1, and 1 mg) with 14 vials all together, followed by 2 boxes of series I (0.1, 1, and 10 mg) with 14 vials; and subsequent treatment with series II (1, 10, and 20 mg) continuously until 12th month. Local reactions at the injection site (redness, slight swelling, and itching) with more than 5 cm diameter were followed by dose reduction, that is, injection of half an ampoule (discarding the rest).

Treatment with Oral Etoposide tablets was done at the dose of 50 mg/m^2^ per day for 21 days, followed by one week rest. This schedule was repeated for 6 cycles. If G3/G4 hematological toxicity occurred, according to the study plan the cycle was shortened to 14 days. If neutrophils were below 500/*μ*L, G-CSF could be administrated until the count reached 1000/*μ*L. If patients experienced G3/G4 toxicity over 2 cycles, total dose of Etoposide was reduced to 50%. Patients experiencing G3/G4 toxicity over the next cycle despite dose reduction were withdrawn from treatment.

The staging examinations performed at screening (month −0.5) and during the study at baseline (month 0) and 3, 6, 9, and 12 months after start of treatment are shown in Table 1.

### 2.4. Endpoints

Primary endpoint of the study was PRDFS after the second relapse in osteosarcoma patients, assessed at each visit by X-ray or computer tomography (CT) of the primary site of the tumor (bone) and CT of the lung and additionally by ultrasound examination or CT of the abdomen after 6 months of treatment. The primary efficacy parameter PRDFS rate was defined as the proportion of patients in a given treatment arm without any sign of relapse after 12 month of treatment with* Viscum* or Etoposide, respectively. As the PRDFS rate without treatment is known to be about 12% from retrospective studies [2, 3], the aim of this study was to assess whether any of the two treatments might have the potential to increase PRDFS rate to about 40% one year after surgery after the second relapse. Patients are followed up beyond the end of the trial and their PRDFS status is updated on an ongoing basis. Until July 2013 follow-up times up to 73 months have been documented.

Second endpoints were the quality of life (QoL) in both arms measured by the core questionnaire of the European Organization for Research and Treatment of Cancer (EORTC QOL-C30) in adults or by the Pediatric Quality of Life Cancer Module Acute Version 3.0 (PedsQL) in patients <18 years, respectively. The EORTC QLQ-C30 consists of 30 questions with four (28 questions) or seven (2 questions) response categories in the form of Likert scales. The questions are subsumed to five functioning scales (physical, role, emotional, cognitive, and social), three symptom scales (fatigue, nausea/vomiting, and pain), six single-item scales (dyspnea, sleep disturbance, appetite loss, constipation, diarrhea, and financial impact), and the global health/quality of life scale that can be regarded as overall QoL index. Regarding the PedsQL, there was only one Etoposide patient with postbaseline data, and therefore this questionnaire is disregarded here.

Safety endpoint of the study was the tolerability of Etoposide and* Viscum* treatments. For this, at each visit patients were asked for adverse events which were registered according to the Common Toxicity Criteria for Adverse Events (CTCAE) and assessed for their relationship to the study medications.

### 2.5. Sample Size Estimation

The sample size necessary to demonstrate a statistical superiority, based on the hypothesis that one or both drugs can improve the historically documented PRDFS rate of 12% up to approximately 35%, was estimated to require 18 patients per arm, assuming an alpha error level 5% and a power of 81%. Based on our experience, we anticipated no dropouts. Due to recruitment failure, the study was terminated early by protocol amendment after the inclusion of 20 patients (11 Etoposide, 9* Viscum*).

### 2.6. Statistical Methods

Comparability between treatment arms was assessed using arithmetic mean, standard deviation, minimum, first, and third quartile, median, and maximum for continuous data, and contingence tables showing absolute and relative frequencies for categorical data. No tests for difference between treatment groups were performed for these baseline variables since *P* values smaller than 5% only represent the expected 1 in 20 chance to find a difference where none exists in reality.

The analysis of the efficacy parameters followed the intention-to-treat approach; that is, all patients were included in the analysis as randomized. All efficacy analyses were done separately for each of the two treatment groups.

The evaluation of the primary endpoint PRDFS rate was performed as comparison of the 12-month PRDFS rate with the fixed value of 12% (i.e., the average PRDFS rate after second relapse in historical control groups) using an exact Binomial test [28].

A linear mixed model was used to analyze the QoL parameters of the EORTC QLQ-C30 as difference to baseline, including the respective baseline value of each QoL parameter, the treatment group and the visit as independent factors, and study patients as random factor. The dependency between successive visits within each patient was accounted for using a compound symmetry covariance matrix.

All tests were performed on an alpha error level of 5%; due to the exploratory character of this trial, no adjustment for multiple testing was applied. Together with the *P* values, 95% confidence intervals are reported.

## 3. Results

From June 2007 to July 2011, 20 patients had been enrolled. Eleven patients were randomly assigned to the Etoposide arm and nine to the* Viscum* arm. Histology confirmed osteosarcoma in all patients; all patients had undergone surgery for a second relapse of the disease in the lung, and two in the hip for local relapse of proximal femur primitive localization. A second chemotherapy had already been applied in 5 (55.5%) (*Viscum* arm) and 4 (36.4%) (Etoposide arm) patients after first relapse, respectively, the last one about three years before entry into the study.

Male to female ratio was 11 : 9; mean age was 33.9 years (range 11–65). Median DFS from first surgery to first relapse and from first to second relapse was 19.1 (2–40) and 21.1 (3–82) months in the* Viscum* arm and 26.9 (14–37) and 15.6 (3–40) months in the Etoposide arm, respectively. Other sociodemographics, disease, and treatment related baseline characteristics are shown in Tables 2 and 3.

**Figure 1:**
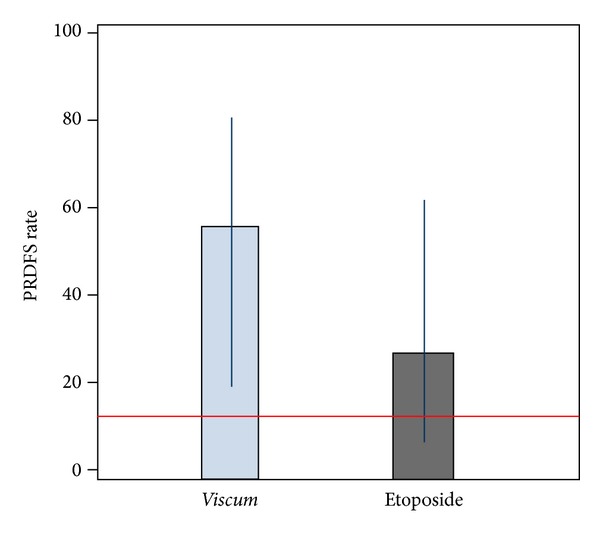
PRDFS rates and exact 95% confidence intervals after 12 months of treatment with* Viscum* or Etoposide, respectively. The horizontal line represents the 12% PRDFS rate derived from historical controls. By crossing the 12% line, the Etoposide confidence interval indicates that this treatment cannot statistically be distinguished from the historical rate, whereas for* Viscum* a significant difference can be deduced.

After one year of treatment PRDFS rate in* Viscum* arm was 55.6%, compared to historical 12% rate: *P* = 0.0041; 95% CI (21.2%; 86.3%) (five out of nine patients) and it was 27.3%, *P* = 0.2724; 95% CI (6.0%; 61.0%) for Etoposide arm (three out of eleven patients) (see Figure 1).

**Figure 2:**
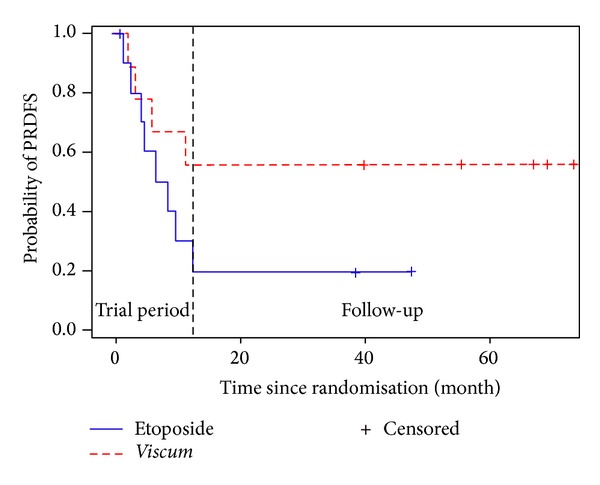
Kaplan-Meier graph of the course of PRDFS for* Viscum* and Etoposide patients, respectively, over the period of the trial and during follow-up. The vertical line indicates the end of the trial period. Last dates are from July 2013 and are updated on an ongoing basis.

Until July 2013 in the* Viscum* arm the median PRDFS (including censored dates) is 39 months (range 2–73 months). One out of 6 patients relapsed locally in the area of previous surgery (pelvis). In the Etoposide arm median PRDFS is 4 months (1–47 months) (see Figure 2). One patient enrolled in the Etoposide arm refused to accept Etoposide after randomization and withdrew from the trial and took* Viscum* instead; nevertheless, following intention-to-treat approach he was analyzed as assigned to Etoposide. Another patient relapsed after three months of Etoposide; after surgery for the third relapsed disease he crossed over to* Viscum* for 2 years. He is still free from disease from his 3rd relapse after 59 months. Two patients in the* Viscum* arm after one year of* Viscum *study treatment decided to continue* Viscum* therapy at least for another year spontaneously.

Regarding the quality of life assessment the trend was positive for* Viscum *treatment (Table 4); this can especially be seen in the global QoL scale, in the functional scales “physical functioning” and “social functioning,” and for the symptom items “fatigue,” “pain,” “dyspnea,” and “financial difficulties” of the EORTC QLQ-C30. A similar improvement under Etoposide could only be seen for “social functioning.” Here, on the other hand, deteriorations had to be observed for “nausea/vomiting” and “pain.”

Regarding the safety of the patients, five Serious Adverse Events (SAE) occurred during the trial due to hospitalization of patients for surgery (2 Viscum, 1 Etoposide patient) and for pneumonia (2 Etoposide patients). Pneumonia was regarded as related to the Etoposide treatment; therefore, these SAEs constitute serious adverse drug reactions (SAR). Regarding further adverse drug reactions (ADR), no toxicity was reported under* Viscum* treatment except negligible local erythema after sc injection and hypotension in one patient. Under Etoposide, observed toxicity included G2, G3 hematologic toxicity (Table 5). G-CSF was necessary in three patients. Two patients needed dose reduction (14 instead of 21 days per cycle) due to hematologic toxicity, and one patient needed blood transfusion for G4 anemia (1 episode).

## 4. Discussion

The treatment of relapsed osteosarcoma patients is unsatisfactory especially after a second or further relapse because there is no effective adjuvant treatment besides surgery that can prolong PRDFS. In addition to this, heavily pretreated patients often do not want to receive another aggressive treatment with serious side effects.

The relationship between cancer and the immune system is well known [29]. Also, in osteosarcoma a relationship between infections as a favorable prognostic factor has been documented [30] and a new trend of immunotherapy as adjuvant treatment is emerging in the therapy of osteosarcoma. Interferon-Alpha (IFN) was used in osteosarcoma in the 1960ies at the Karolinska Institute before the chemotherapy era, and 10-year overall survival (OS) results are similar to those attained with chemotherapy alone [31]. Muramyl tripeptide (MTP) is a BCG derived drug with immunomodulating activity activating macrophage tested at Memorial Sloan Kettering Cancer Center together with chemotherapy with improved DFS and prolonged overall survival. A significant gain in OS from 70% to 78% could be observed after 6 years of follow-up [32].

IFN and MTP are quite expensive. MTP is reimbursed by the Italian health system only for the adjuvant treatment of high risk nonmetastatic osteosarcoma patients (<30 years old) together with chemotherapy, at a high cost (total treatment of 6 months is about € 100.000).* Viscum album fermentatum* has a long history being used for over 80 years; its toxicity is well known and its costs are much more affordable compared to the other two drugs.

Of course our study has major drawbacks. The interpretation of its results is limited by the low number of patients treated, and a larger study is needed for confirmative proof of these preliminary findings. Also, the use of* Viscum album fermentatum Pini* is based on the manufacturer's recommendations for the treatment of sarcomas, which do not include a rationale in this regard [33]. The chosen preparation may be remarkable since the pharmacological effects of mistletoe extracts have to a large extent been attributed to mistletoe lectins [34–36], and other mistletoe extracts of this manufacturer exceed* Viscum album fermentatum Pini* regarding their lectin contents by a factor ranging between 15 and 35. Yet, a pine mistletoe extract was shown to be more potent in enhancing the activity of lymphocytes compared to another extract (Iscador M) richer in mistletoe lectin content [37]. So either the efficacy of mistletoe extract is not (only) depending on the amount of mistletoe lectins or pharmacologically active principles other than mistletoe lectins contribute in a relevant way. Indeed, viscotoxins [36, 38, 39] have been acknowledged as pharmacologically active substances, and other constituents like Kuttan peptides [40], quercetin [41, 42], and polysaccharides [17, 18] have shown antitumor or immunomodulatory activities* in vitro*.

Regardless of these unresolved issues, so far the results indicate a positive trend in PRDFS for* Viscum* compared to historical control, and descriptively also compared to Etoposide. Moreover,* Viscum* patients tend to remain superior to Etoposide patients in several domains of their quality of life. Even if this study has an open-label design and the subjective assessment of QoL may be influenced by the patients knowing about their actual treatment, it seems doubtful that this knowledge unduly affects the patients' QoL assessments; rather, it is more likely that the lower quality of life in Etoposide patients is associated with the higher frequency and intensity of adverse drug reactions observed for this treatment.

## 5. Conclusions

Therapy with* Viscum* seems to be a promising adjuvant treatment in prolonging DFS of patients free from disease after their second relapse. Etoposide does not seem to prolong DFS. A larger study in this subgroup of patients might be of value, which might compare* Viscum* with other immunomodulators like IFN or MPT.

## Figures and Tables

**Table 1:** Schedule of events over the study.

Month	−0.5	0	1	2	3	6	9^a^	12^a^
Informed consent	X							
Inclusion/exclusion criteria	X							
Medical history/adverse events	X	X	X	X	X	X	X	X
Physical examination*	X	X	X	X	X	X	X	X
QoL (EORTC/POQOL)	X	X			X	X	X	X
CBC, biochemical profile**	X					X		X
Urine analysis	X							X
Pregnancy test***	X							
CT Lungs	X				X	X	X	X
Bone X-ray or CT					X	X	X	
Total bone scan	X							X
Ultrasound/CT abdomen	X					X		X
Medication dispense		X	X	X	X	X	X	
Return of unused medication			X	X	X	X	X	X
Immunological evaluation		X			X	X	X	X

*Full PE on month −0.5 and on exit visit; examination of disease-related findings only at other visits.

**Within two weeks prior to screening or within baseline period.

***For premenopausal women.

^a^Treatment duration for *Viscum*: 12 months; for Etoposide: 6 months.

**Table 2:** Sociodemographic and general health characteristics.

Patient characteristics	Frequency (percentage) or mean (range)	
	*Viscum n* = 9	Etoposide*n* = 11
Gender		
Male	4 (44.4)	7 (63.6)
Female	5 (55.6)	4 (39.4)
Age (years)	28 (18–48)	39 (11–66)
Ethnic group		
Caucasian	8 (88.9)	11 (100)
Asian	1 (11.1)	—
Family status		
Single/divorced	8 (88.9)	6 (54.5)
Married/in partnership	1 (11.1)	5 (45.5)
Highest education		
Vocational training	5 (55.6)	7 (63.6)
University graduate/student	4 (44.4)	4 (36.5)
ECOG		
0	3 (33.3)	6 (54.6)
1	6 (66.7)	5 (45.4)
≥2	—	—
Concomitant diseases	1 (11.1)	3 (27.3)
Paget syndrome	—	1 (11.1)
HCV	1 (11.1)	—
Primary hyperparathyroidism	—	1 (11.1)
Kidney tubulopathy	—	1 (11.1)
Current regular medication	5 (55.6)	6 (54.6)
Current signs and symptoms		
Pain	2 (22.2)	1 (9.1)
Weight loss	2 (22.2)	1 (9.1)
Cough	1 (11.1)	1 (9.1)
Dyspnea	1 (11.1)	—

**Table 3:** Disease and treatment specific baseline characteristics.

Tumor disease characteristics	Frequency (percentage) or mean (min–max)	
	*Viscum n* = 9	Etoposide* n* = 11
Time since primary diagnosis (years)	4.0 (1.5–10.5)	3.7 (1.4–7.2)
DFS 1° interval (months)	22.3 (2.9–43.3)	27.9 (14.5–39.4)
DFS 2° interval (months)	22.9 (3.0–82.1)	14.9 (1.8–47.4)
Time since 2° relapse (weeks)	13.9 (0.9–76.6)	7.6 (1.9–24.6)
Osteosarcoma		
Chondrosarcomatous	1 (11.1)	2 (18.2)
Osteoblastic	4 (44.4)	5 (45.5)
Spindle cell sarcoma	0	1 (9.1)
Not otherwise specified	4 (44.4)	3 (27.3)
Staging (Enneking)		
I (I B)	0	1 (9.1)
II (II A, II B)	6 (66.7)	8 (72.7)
III (III, III A, III B)	3 (33.3)	2 (18.2)
Grading		
2	8 (88.9)	11 (100)
3	1 (11.1)	—
4	—	—
Metastases present	9 (100)	11 (100)
2nd chemotherapy after 1st relapse	5 (55.6)	4 (36.4)
Time since last chemotherapy (years)	3.0 (0.6–10.5)	2.8 (0.4–7.2)
Radiotherapy	—	—
Frequency of surgeries		
3	5 (55.6)	9 (81.8)
4	3 (33.3)	1 (9.1)
5	1 (11.1)	1 (9.1)
Time since last surgery (months)	1.5 (0.7–2.0)	2.2 (1.2–5.9)

**Table 4:** Mean changes from baseline for the QoL scales of the EORTC QLQ-C30.

EORTC QLQ-C30 scale	Estimated changes*	95% CI	*P*-value
Physical functioning			
* Viscum *	7.30	[0.15; 14.44]	0.046
Etoposide	−2.45	[−8.93; 4.03]	0.430
Role functioning			
*Viscum *	3.80	[−7.94; 15.54]	0.827
Etoposide	−6.31	[−18.28; 5.65]	0.508
Emotional functioning			
*Viscum *	−5.98	[−10.58; −1.37]	0.014
Etoposide	−2.48	[−9.84; 4.87]	0.481
Cognitive functioning			
*Viscum *	−0.92	[−6.49; 4.65]	0.734
Etoposide	−5.94	[−12.19; 0.31]	0.061
Social functioning			
*Viscum *	11.76	[4.64; 18.88]	0.003
Etoposide	4.78	[0.51; 9.05]	0.031
Global health/QoL			
*Viscum *	11.17	[2.62; 19.72]	0.013
Etoposide	3.51	[−3.51; 10.54]	0.301
Fatigue			
*Viscum *	−9.85	[−16.31; −3.38]	0.005
Etoposide	1.13	[−5.72; 7.99]	0.73
Nausea/vomiting			
*Viscum *	0.43	[−2.70; 3.56]	0.779
Etoposide	5.47	[0.28; 10.66]	0.040
Pain			
*Viscum *	−10.71	[−18.83; −2.60]	0.012
Etoposide	10.54	[4.64; 16.45]	0.002
Dyspnoea			
*Viscum *	−12.63	[−16.94; −8.32]	<0.0001
Etoposide	5.82	[−1.04; 12.68]	0.090
Insomnia			
*Viscum *	−11.35	[−20.74; −1.96]	0.020
Etoposide	5.79	[−2.95; 14.53]	0.177
Appetite loss			
*Viscum *	−6.40	[−6.40; −6.40]	N.E.^†^
Etoposide	1.41	[−2.15; 4.96]	0.410
Constipation			
*Viscum *	−5.54	[−13.58; 2.50]	0.166
Etoposide	−0.62	[−9.65; 8.41]	0.884
Diarrhea			
*Viscum *	0.83	[−2.81; 4.47]	0.639
Etoposide	2.44	[−1.92; 6.80]	0.251
Financial problems			
*Viscum *	−11.46	[−16.21; −6.70]	<0.0001
Etoposide	−2.53	[−6.88; 1.83]	0.234

*Estimates resulting from a linear mixed model, including baseline score, treatment and visit as fixed factors, and patients as random factors.

^†^All postbaseline values in the *Viscum* group were 0; therefore no test statistic could be calculated.

**Table 5:** Frequency and intensity of adverse events (AE) and adverse drug reactions (ADR).

AE characteristics	*Viscum N* [%]	Etoposide *N* [%]	Total *N* [%]
All AEs	16 [18.8]	69 [81.2]	85 [100.0]
Unfavorable AEs by			
Severity			
Severe	5 [5.88]	26 [30.59]	28 [36.47]
Outcome			
AE unchanged	4 [4.76]	4 [4.76]	8 [9.52]
AE exacerbated	—	2 [2.38]	2 [2.38]
Study medication			
Dose reduced	—	5 [5.88]	5 [5.88]
Use continued after interruption	1 [1.18]	18 [21.18]	19 [22.35]
Use discontinued	2 [2.35]	14 [16.47]	16 [18.82]
Adverse drug reactions (ADR)	2 [2.36]	47 [55.29]	49 [57.65]
Most frequent ADR			
Neutropenia	—	12 [25.53]	12 [24.49]
Anaemia	—	6 [12.77]	6 [12.24]
Leukopenia	—	6 [12.77]	6 [12.24]
Nausea	—	5 [10.64]	5 [10.20]
Alopecia	—	4 [8.51]	4 [8.16]
